# The complete chloroplast genomes of two species from Capparaceae

**DOI:** 10.1080/23802359.2020.1852902

**Published:** 2021-02-20

**Authors:** Dhafer Alzahrani, Enas Albokhari, Samaila Yaradua, Abidina Abba

**Affiliations:** a Department of Biological Sciences, Faculty of Science, King Abdulaziz University, Jeddah, Saudi Arabia; b Department of Biological Sciences, Faculty of Applied Sciences, Umm Al-Qura University, Makkah, Saudi Arabia

**Keywords:** *Capparis spinosa*, *Capparis decidua*, Capparaceae, chloroplast genome

## Abstract

*Capparis spinosa* L. and *Capparis decidua* Forsk. belong to the Capparaceae family. The two species are important medicinal plants uses in treatment of various ailments. In this study, we present the complete chloroplast genomes of the two species. The complete plastome genomes of the two species have a circular structure and a length of 157,728 bp in *Capparis spinosa* and 157,573 bp in *Capparis decidua* and GC content of 35.91, 35.96% respectively. The chloroplast genome of *C. spinosa* and *C. decidua* is divided into four regions: LSC of 86,732 and 85,950 bp respectively, SSC from 18,322 to 18,621 bp and a pair of inverted repeats 26,337 and 26,501 bp each. Both of the chloroplast genomes contained 115 different genes, including 80 protein coding genes, 31 tRNA genes and four rRNA genes. A phylogenetic analysis demonstrated that *C*. *spinosa* is sister to *Capparis urophylla*. The two species are sister to *C. decidua*.

## Introduction

The family Capparaceae Juss. is also known as Capparidaceae and it is comprised of ca. 17 genera and approximately 470 species (Chayamarit 1991; Fay and Christenhusz 2010). Some of the species in the family are used as ornamentals while others are used in traditional medicine especially members of the genus *Capparis*. *Capparis* is one of the largest genera of the subfamily Capparoideae, consisting of ca. 139 species, distributed in tropical and subtropical regions of the Old World (POWO 2019). *Capparis* is an important medicinal plant that has a wide range of bioactive compounds such as alkaloids, flavonoids, streroids, terpenoids, and tocopherols. The species are used in the treatment of various ailments such as the treatment of diabetes, high blood pressure, rheumatism, arthritis and liver, spleen and kidney disorders and are used as antifungal agent, antioxidant and anti-inflammatory and have odynolytic properties (Baytop 1984; Nijveldt et al. 2001; Taifour et al. 2011).

One of the most important factors that help in the study of molecular evolution, population genetic studies and taxonomy is chloroplast (cp) DNA genome. In this current study, we present the assembled sequence of the complete chloroplast cp genomes of two *Capparis* species: *Capparis spinosa* (MT041701) and *Capparis decidua* (MT948186) for the purpose of providing bases of genetic information for future research.

In this study, fresh leaves of *Capparis spinosa* were collected from Jeddah, Saudi Arabia (21°26′45″N 39°25′22.9″E) and *Capparis decidua* were collected from Rabigh, Saudi Arabia (22°48′31.1″N 39°02'08.9″E). Voucher specimens were prepared and deposited in the herbarium of King Abdulaziz University, Jeddah with the accession number (*Capparis spinosa* KAU27390 and *Capparis decidua* KAU27391). DNA extraction was done using Qiagen DNA extraction kit (Qiagen, Hilden, Germany) based on manufacturer’s guidelines. The Extracted DNA was sequenced using Illumina Hiseq 2500 platform (Novogene Technology, Inc. Beijing, China) with the average read length of 150 pb. Raw data were filtered using PRINSEQ lite Ver0.20.4 to obtain clean reads (5GB). Trimmed sequences were assembled with NOVOPlasty (Dierckxsens et al. 2017). The assembled sequences were annotated using DOGMA (Wyman et al. 2004) which adopt the manual adjustment using BLAST (https://blast.ncbi.nlm.nih.gov/Blast.cgi), trNAscan-SE2.0 (Lowe and Chan 2016) was used to identify tRNA genes. Finally, the complete chloroplast genome sequences of both species were submitted to the GenBank with the accession number *C. spinosa:* MT041701; *C. decidua*: MT948186.

The complete plastome genome of *C. spinosa* is 157,728 bp in length and *C. decidua* is 157,573 bp. Both of the cp genomes have a circular topology with GC content of 35.91% GC content in *C. spinosa* and 35.96% in *C. decidua*. Both species have quadripartite structure: two inverted repeats (IRs), a large single copy region (LSC), and a small single copy region (SSC). Large single copy (LSC) ranged from 86,732 bp (*C. spinosa*) to 85,950 bp (*C. decidua*) and small single copy SSC ranged from 18,322 bp (*C. spinosa*) to 18,621 bp (*C. decidua*). The length of IR in *C. spinosa* is 26,337 bp while that of *C. decidua* is 26,501 bp. Each of the two genomes contained 80 protein coding genes, 31 tRNA genes and 4 rRNA genes. Out of the 115 genes, 12 genes contained one intron and two genes contained two introns in *C. spinosa* and 13 genes contained one intron and two genes contained two introns in *C. decidua*.

To evaluate the phylogenetic position of both species of *Capparis*, a complete chloroplast genome of three species from Capparaceae were downloaded from the GenBank: *Capparis versicolor* NH142726, *Crateva unilocularis* MT679554 and *Capparis urophylla* CNA0000813. Three species from Brassicaceae (*Cakile arabica* NC_030775, *Lepidium sativum* NC_047178 and *Cardamine macrophylla* MF405340) were also downloaded from GenBank to be used as outgroup.

**Figure 1.**
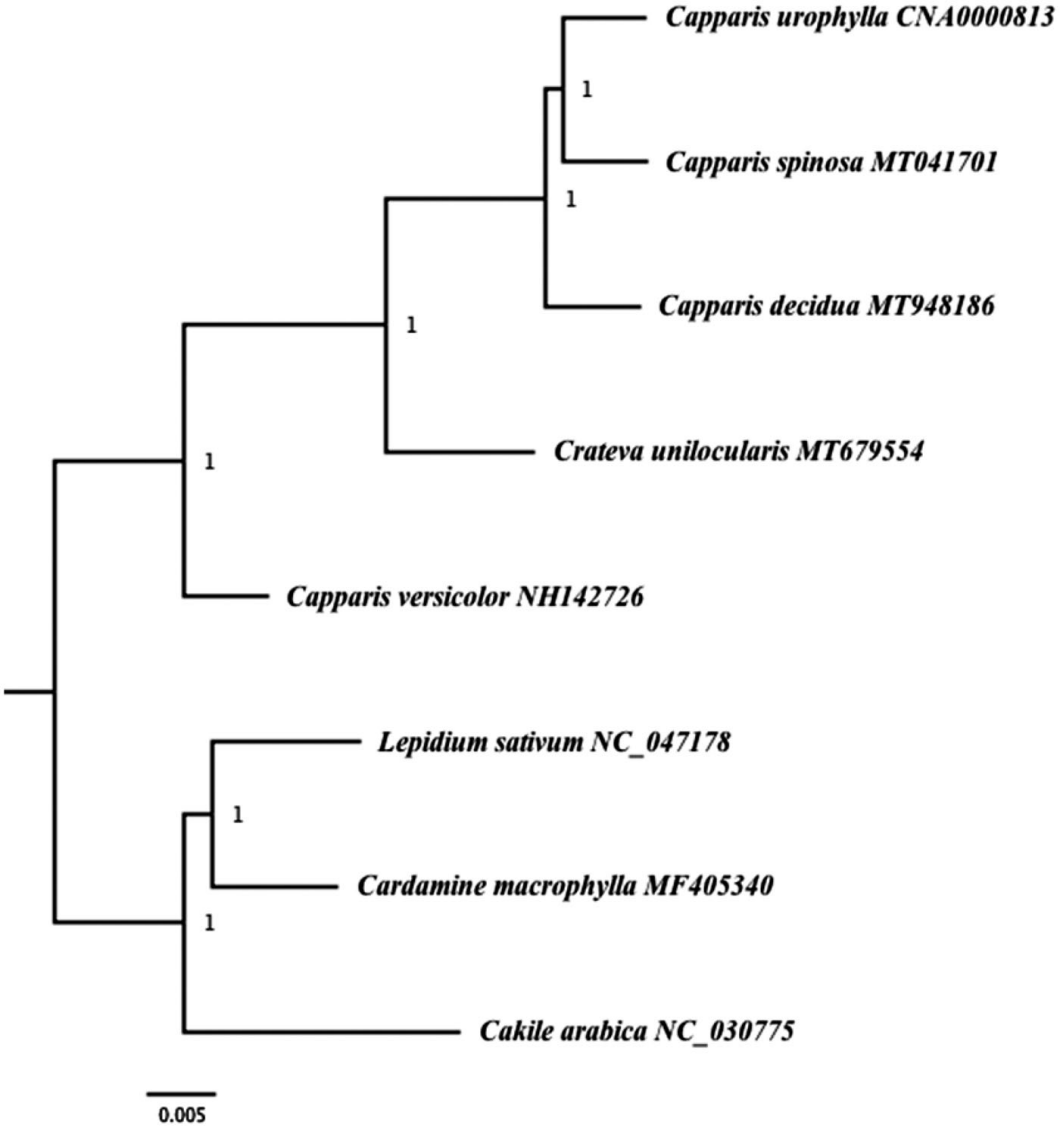
Bayesian Inference (BI) phylogenetic tree of two species of *Capparis* and other species based on complete chloroplast genome sequence. Numbers in the nodes represent posterior probability (PP) values.

The downloaded sequences and the sequenced cp genomes were aligned using MAFFT (Katoh and Standley 2013) and the phylogenetic tree was constructed using Maximum Parsimony method using PAUP version 4.0b10 (Felsenstein 1978) using heuristic searches with 1000 replicates of random taxon addition, tree bisection– reconnection branch swapping, MulTrees on, saving a maximum of 100 trees each replicate. Missing characters were treated as gaps. Support was assessed using 1000 replicates of non-parametric bootstrap analysis. The result of the phylogenetic analysis showed that *C*. *spinosa* is sister to *Capparis urophylla* and the two species are sister to *C. decidua* (Figure 1).

## Data Availability

The data that support the findings of this study are openly available in GenBank of NCBI at https://www.ncbi.nlm.nih.gov, reference number (*C. spinosa:* MT041701; *C. decidua*: MT948186).
